# Protective effect of Hibiscus *sabdariffa* against serum/glucose deprivation-induced PC12 cells injury

**Published:** 2015

**Authors:** Elham Bakhtiari, Azar Hosseini, Seyed Hadi Mousavi

**Affiliations:** 1 *Department of Pharmacology, School of Medicine, Mashhad University of Medical Sciences, Mashhad, Iran*; 2 *Neurocognitive Research center, School of Medicine, Mashhad University of Medical Sciences, Mashhad, Iran.*; 3 *Pharmacological Research Center of Medicinal Plants, School of Medicine, Mashhad University of Medical Sciences, Mashhad, Iran.*

**Keywords:** *Hibiscus sabdariffa*, *SGD*, *PC12 cell*, *Apoptosis*

## Abstract

**Objectives:**

Findings natural products with antioxidant and antiapoptotic properties has been one of the interesting challenges in the search for the treatment of neurodegenerative diseases including ischemic stroke. Serum/glucose deprivation (SGD) has been used as a model for the understanding of the molecular mechanisms of neuronal damage during ischemia in vitro and for the expansion of neuroprotective drugs against ischemia-induced brain injury. Recent studies showed that *Hibiscus sabdariffa* exert pharmacological actions such as potent antioxidant. Therefore, in this study we investigated the protective effect of extract of *H. sabdariffa* against SGD-induced PC12 cells injury.

**Materials and Methods:**

Cells were pretreated with different concentrations of *H. sabdariffa* extract (HSE) for 2 hr, and then exposed to SGD condition for 6, 12 and 18 hr.

**Results:**

SGD caused a major reduction in cell viability after 6, 12, and 18 hr as compared with control cells (p< 0.001). Pretreatment with HSE (30-500 𝜇g/mL) significantly increased cell viability following SGD insult for 6, 12 and 18 hr. A significant increase in cell apoptosis was seen in cells under SGD condition after 12hr as compared with control cells (p< 0.001). Pretreatment with HSE significantly decreased cell apoptosis subsequent SGD conditionafter12hr at concentration of 60, 125 and 250.

**Conclusion:**

These data showed that HSE had a protective property under SGD condition in PC12 cells, suggesting that *H. sabdariffa* has the potential to be used as a new therapeutic approach for neurodegenerative disorders.

## Introduction

Despite significant progress in the treatment of cerebral ischemia, stroke still remains one of the most important causes of death in populations (Amantea et al., 2009 ▶). Because the fundamental pathophysiology of stroke is decrease of glucose, O_2_, and other nutrients toward neurons, SGD can be used as a suitable model to assess stroke process (Moley and Mueckler, 2000 ▶). Designing the neuroprotective agents could be done with an efficient in vitro model such as SGD neuronal damage which could describe the molecular mechanism of brain injury during cerebral ischemia (Chu al., 2008 ▶; Hillion et al., 2005 ▶). PC12 rat pheochromocytoma cell line has been widely utilized as in vitro model to investigate the SGD condition and other alterations in neural tissue (Reimann-Philipp et al., 2001 ▶; Woronowicz et al., 2007 ▶).

*Hibiscus sabdariffa* (also famous as roselle and sour tea) from the Malvaceae family is cultured and grown naturally in tropical and subtropical regions, including south of Iran. The plant has been used in different countries as a culinary and medicinal substance. The fat fruiting calyces of this plant, which are sour in taste, have been used for preparing candies and beverages. In traditional medicine, the calyx extract of this plant is used for treatment of several diseases, including high blood pressure (Ali et al., 2005 ▶), liver diseases (Ali et al., 2005 ▶), cardiovascular diseases (Chen et al., 2004 ▶), and atherosclerosis (Chen et al., 2004 ▶). The chemicals existing in the flowers of *H. sabdariffa *is including tartaric acid, malic and citric acid, anthocyanins (delphinidin-3-glucoxyloside, delphinidin- entoside-glucoside, delphinidin-3-ambubioside, cyanidin-monoglucoside, cyanidin-3,5-diglucoside, cyanidin-3-sambubioside, cyanidin-3-glucosylrutinoside, and cyanidin-3-glucoside), flavonol glycoside, gossypitrin, sabdaretin, myricetin, hibiscetrin, quercetin, luteolin, a luteolin glycoside and chlorogenicacid, flavonoids (gossypetin, hibiscetin, and their respective glycosides), sterols (*β*-sitosterol and ergosterol), and protocatechuic acid. Diverse strains of *H. sabdariffa* from different countries may vary in one or several of these constituents (Ali et al., 2005 ▶). Almost all of these chemical ingredients have potent antioxidant properties. Several studies reported that *H. sabdariffa* extract has potent antioxidant properties and has great capacity for scavenging free radicals (Tseng et al., 1997 ▶; Oboh and Rocha, 2008 ▶).

As it has been revealed that *H. sabdariffa *has many valuable properties correlating with its antioxidant effect, the aim of this study was to examine the cytotoxic effect of HSE against the SGD-induced PC12 cells injury.

## Material and Methods

**Cell line and substances**

PC12 cell line was purchased from Pasteur Institute (Tehran, Iran). 4, 5-dimethylthiazole-2-yl, 2, 5-diphenyl tetrazolium (MTT) and Dulbecco’s phosphate-buffered saline (PBS) were purchased from Sigma (St Louis, MO, USA). Glucose-high Dulbecco’s modified Eagle’s medium (DMEM), Glucosefree DMEM, fetal bovin serum (FBS), and penicillin streptomycin were purchased from Gibco (Grand Island, NY). Dimethyl sulfoxide (DMSO) was purchased from Merck. Propidium iodide (PI), sodium citrate and Triton X-100 were purchased from Sigma (St. Louis, MO, USA).

**Cell culture**

PC12 cells were cultured in high glucose DMEM (4.5 g/L) supplemented with 10% FBS and 100 unit/mL of penicillin/streptomycin. All cells were maintained in a humidified atmosphere (90%) containing 5% CO2 at 37^0^C.

**Induction of cell death by serum/glucose deprivation**

For SGD-induced cytotoxicity, PC12 cells were seeded overnight and then were exposed to SGD for 6, 12, and 18 hr by switching the standard culture medium (high glucose DMEM, 4.5 g/L) with the glucose-free DMEM (0 g/L), supplemented with 100 U/mL penicillin and 100 U/mL streptomycin (Mousavi et al., 2010 ▶).

**Preparation of** ***hibiscus sabdariffa*** **extract (HSE)**

Whereas *H. sabdariffa* is not native to IRAN, it was afforded from a local herb

market in Mashhad and confirmed by plant specialist of Ferdowsi University of Mashhad (FUMH). The calyces of *H. sabdariffa* were dried, powdered and subjected to extraction with 70% ethanol in a Soxhlet apparatus for 48 hr. The HSE extract was then dried on a water bath and the yield (24% w/w) dissolved in DMSO.

**Cell proliferation (MTT) assay**

PC12 cells (5000/well) were seeded out in 96-well culture plate, and after 24 hr the cells were pretreated with HSE (30-500 𝜇g/mL) for 2 hr and then incubated simultaneously for another 6, 12 and 18hr in SGD condition. MTT solution in phosphate-buffered saline (5 mg/ml) was added to each well at final concentration of 0.05%. After 3 hr, the formazan precipitate was dissolved in DMSO. The absorbance at 570 and 620 nm (background) was measured using a Stat FAX303 plate reader. All treatments were carried out in triplicate.

**Cell apoptosis assay**

Apoptotic cells were detected using PI staining of small DNA fragments followed by flow cytometry. It has been reported that a sub-G1 peak that is reflective of DNA fragmentation can be observed following the incubation of cells in a hypotonic phosphate-citrate buffer containing a quantitative DNA-binding dye, such as PI. Apoptotic cells that have lost DNA take up less stain and appear on the left side of the G1 peak in the histogram. Briefly, PC12 cells were seeded in wells of a 24-well plate and after 24 hr the cells were pretreated with HSE (30-500 𝜇g/mL) for 2 hr and then incubated simultaneously for 12 hr in SGD condition. Floating and adherent cells were then harvested and incubated at 4 °C overnight in the dark with 750 µl of a hypotonic buffer (50 µg/ml PI in 0.1% sodium citrate with 0.1% Triton X-100). Next, flow cytometry was carried out using a FACScan flow cytometer (Becton Dickinson). A total of 104 events were acquired with FACS. All treatments were carried out in triplicate.

**Statistical analysis**

One-way analysis of variance (ANOVA) followed by Tukey’s post hoc test for multiple comparisons were used for data analysis. All results were expressed as mean *±* SEM. *P <*0*.*05 was considered statistically significant.

## Results

**HSE dose-dependently inhibits SGD-induced cell death**

To study the possible toxic effects of HSE, PC12 cells were incubated with different concentrations of HSE (30-500 𝜇g/mL), and the viability was determined 6, 12 and 18 hr after treatment. No significant toxic effect on cell viability was seen subsequent to treatment with HSE for 6, 12 and 18 hr.

**Figure 1:**
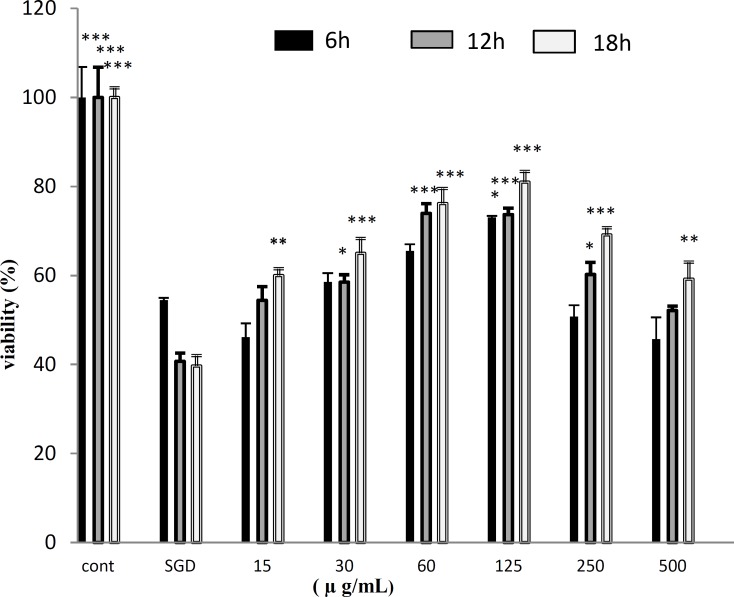
Effect of HSE on PC12 cells viability exposed to SGD (serum/glucose deprivation) for 6, 12 h and 18 hr. The percentage cell viability (quantitated by MTT assay) was normalized against the control. *** *P*<0.001, ** *P*<0.01, * *P*<0.05. Data are expressed as Mean±SEM of three separate experiments (n=3).

SGD caused a significant reduction in cell viability after 6, 12 and 18 hr, as compared with control group. As shown in Figure 1, treatment with HSE resulted time and concentration dependent increase cell viability subsequent to ischemic insult for 6 hr (p<0.05 at concentration of 125 𝜇g/mL) 12 hr (p<0.05 at concentration of 30 𝜇g/mL and 250 𝜇g/mL, p<0.001 at concentration of 60-125 𝜇g/mL) and 18hr (p< 0.01 at 500𝜇g/mL and 15 𝜇g/mL, p< 0.001 at concentration of 30-250 𝜇g/mL).

**HSE significantly decreases SGD-induced cell apoptosis**

**Figure 2:**
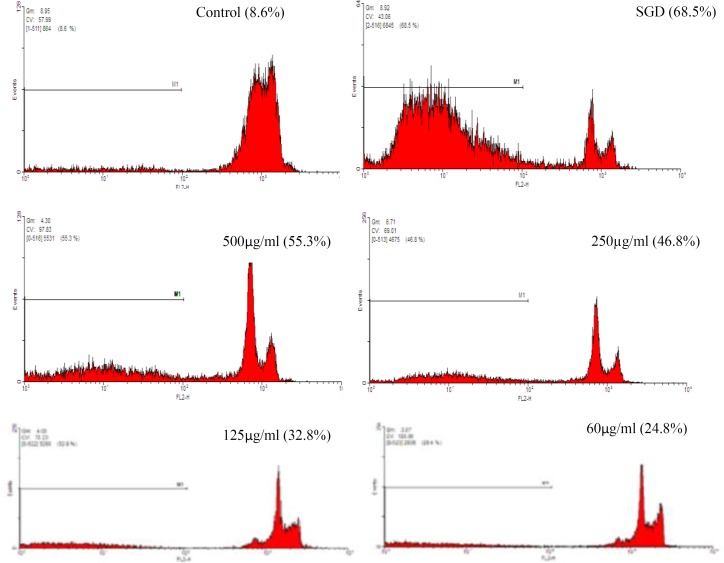
The effects of the HSE on apoptosis in PC12 cells using PI staining and flow cytometry

**Figure 3:**
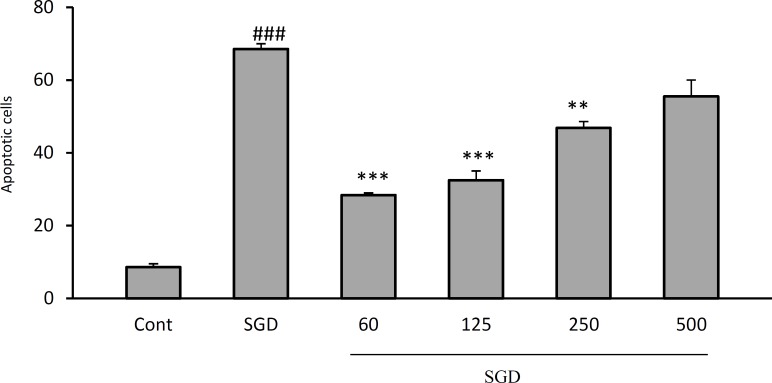
The effects of the HSE on apoptosis in PC12 cells using PI staining and flow cytometry. ^###^*p*< 0.001 versus control, ^**^ p < 0.01 and ^***^ p < 0.001 versus SGD.

The results showed that exposure of PC12 cells to SGD, significantly increased cell apoptosis, compared with control group (p<0.001, Figure 2, 3).

A significant reduction in SGD-induced apoptosis was seen following pretreatment with HSE (60𝜇g/ml, p< 0.001; 125 𝜇g/ml, p< 0.001; 250 𝜇g/ml, p< 0.001 and 500 𝜇g/ml, p<0.001).

## Discussion

 Ischemic stroke is the third leading cause of death and disability in developed countries. Currently, therapeutic choices for treatment of stroke are restricted. Therefore ectensive efforts are being made to recognize new neuroprotective agents with anti-apoptotic properties (Rosamond et al., 2007 ▶).

Oxidative damage and cell apoptosisare typical features of neurodegenerative diseases such as ischemic stroke (Forouzanfaret al., 2013 ▶). Since the antioxidants scavenge free radicals and reduce oxidative damage, they are probably beneficial for ischemic brain injury treatment. Studies have shown that most of herbs contain effective agents. Some of these agents possess compounds which have anti-oxidant activity. Some of herbs were used in SGD model study and showed significant effects in reducing oxidative damage. Forouzanfar et al. (2013) ▶ reported that *Punica granatum *had protective effect against SGD-induced cytotoxicity (Forouzanfaret al., 2013 ▶). Furthermore, Afsharzadeh investigated the protective effect of *Scutellaria litwinowii* extract against SGD condition and showed that *S. litwinowii* had capacity for scavenging free radicals and could protect the PC12 cells under SGD condition (Afsharzadehet al., 2012 ▶). In this study, the protective effect of *H. sabdariffa *against SGD- induced cell death was investigated in PC12 cells for the first time. Results showed that *H. sabdariffa *has no cytotoxicity on PC12 cells at 30-500 𝜇g/ml concentrations. In this study, up to 50% of cell loss was seen under SGD condition for 6, 12 and 18 hr, which is in agreement with earlier reports (Mousavi et al., 2010 ▶; Lorenz et al., 2009 ▶). Moreover, results showed that pretreatment with *H. sabdariffa* extract significantly increased cell viability and decreased cell apoptosis under SGD condition. Recent studies have shown that *H. sabdariffa *has bioactive properties that may play a fundamental role in preventing chronic diseases such as hypertension, cardiovascular disease, diabetes, atherosclerosis and reduction of high cholesterol (Sindiet al., 2014 ▶). Moreover, *H. sabdariffa *has potent antioxidant properties. It contains anthocyanins, polyphenols and flavonoids which have antioxidant effects (khaghaniet al., 2011 ▶). The antioxidant mechanism of HSE is due to scavenging reactive oxygen and free radicals, inhibition of xanthine oxidase activity, reduction of lipid peroxidation and elevation of antioxidant enzymes activity (Costa-Rocha et al., 2014 ▶). In vitro and in vivo studies have shown antioxidant effects of *H. sabdariffa*. It reduced oxidative stress in rat primary hepatocytes and scavenged free radicals (Tseng et al., 1997 ▶). Moreover, Oboh reported that *H. sabdariffa *has protective effect against pro-oxidant-induced lipid peroxidation in isolated brain of rat (Oboh and Rocha, 2008 ▶). Above mentioned beneficial effects of *H. sabdariffa* were observed for both ethanolic and water extracts from flowers, leaves or seeds (Costa-Rocha et al., 2014 ▶).Therefore the protective activity of *H. sabdariffa *against SGD is through diminution of oxidative damage. In this study, HSE increased cell viability against SGD-induction and decreased cell apoptosis in PC12 cell line possibly via anti-ioxidant activity. Recent studies have shown that HSE makes apoptosis by p38 MAPK and JNK stimulation and translocation of cytochrome c from the mitochondria to the cytosol and caspase cascade activation (Lin et al., 2007 ▶). 

Polyphenol-rich HSE induces apoptosis in gastric carcinoma cells by activation of p38/Jun/FasL signaling and steadying of p53, causing a rise in Bax and cytochrome c release, leading to the activation of caspase-3 (Lin et al., 2005 ▶). *H. sabdariffa* anthocyanin-rich extract induces apoptosis in promyelocytic leukemia cells by augmented phosphorylation of p38 and cytochrome c release, and expression of tBid, Fas, and FasL (Chang et al., 2005 ▶). However, observed apoptotic in PC12 cell line could relate to mentioned mechanisms which need more investigation.

We conclude that *H. sabdariffa *has protective effects against SGD-induced cytotoxicity in PC12 cells. These effects act possibly through its antioxidant activity and antiapoptotic properties. However more researches are required to clarify the probable underlying mechanisms of these useful effects.
